# Continuous Electroencephalogram Monitoring in the Intensive Care Unit

**DOI:** 10.7759/cureus.83444

**Published:** 2025-05-04

**Authors:** Maria Papaioannou, Georgia Vasileiadou, Vasiliki Soulountsi, Anastasia Dimaki, Anastasia Bikouli, Athina Lavrentieva

**Affiliations:** 1 Intensive Care Unit, George Papanikolaou General Hospital of Thessaloniki, Thessaloniki, GRC

**Keywords:** antiepileptic medications (aeds), brain injury, continuous electroencephalogram (ceeg), monitoring, outcome, prognosis, prognostic tool, seizures

## Abstract

Objective: In recent years, continuous electroencephalogram (cEEG) monitoring has become a noninvasive tool for detecting and evaluating brain activity in critical care patients. The study aimed to evaluate the impact of cEEG on the management of comatose intensive care unit (ICU) patients and its association with disease prognosis.

Methods: This observational, prospective study included adult patients in a single general ICU. The study analyzed patients' demographic characteristics, illness severity using the APACHE II (acute physiology and chronic health evaluation) and SOFA (sequential organ failure assessment) scores, comorbidities, reasons for undertaking EEG, EEG patterns, and therapeutic strategies used. Continuous EEG patterns were correlated with patients' outcomes.

Results: Data from 55 patients were analyzed, with a median age of 61 years (range 19-86), a median APACHE II score of 22 (range 5-38), and a median SOFA score of 9 (range 4-16) on admission. The median duration of mechanical ventilation was 35 days, and the median ICU length of stay (LOS) was 42 days. The majority of cEEGs (61.1%) were performed due to medical reasons (ischemic stroke, septic shock, status epilepticus); 25.9% of cEEGs were conducted in neurosurgical patients with post-traumatic acute brain injury and malignancy, whereas 13% were carried out in post-anoxic comatose patients. Patients who started antiepileptic therapy after EEG examination had a higher mortality rate than patients who had already received antiepileptic drugs (AED) (x^2^=7.9565, p=0.004). In more than half of the patients, an encephalopathic EEG pattern was observed in comparison with a lower percentage of patients who had lateralized periodic discharges (LPDs); one patient had burst suppression, and one patient had electrocerebral inactivity (ECI) or silence (ECS). Patients with epileptic disorders had a higher mortality rate (p=0.018) in comparison with the other categories of patients. Taking their medical comorbidities into consideration, patients diagnosed with diabetes mellitus were more likely to have higher mortality (x^2^=5.115, p=0.024).

Conclusion: The management of critically ill patients is influenced by cEEG, which could modify therapeutic strategies and appears to be a useful prognostic tool in critical care patients.

## Introduction

This article was previously presented as a meeting abstract [1] at the 43rd International Symposium on Intensive Care & Emergency Medicine on March 19, 2024.

Continuous electroencephalogram (cEEG) monitoring provides dynamic information about brain function that can detect changes in the neurologic status of comatose critical care patients early. EEG monitoring in the intensive care unit (ICU) is recommended to identify nonconvulsive seizures (NCS) and nonconvulsive status epilepticus (NCSE) in critically ill patients [2] with altered mental status when there is a prior history of epilepsy, unstable level of consciousness, acute brain injury, and recent convulsive status epilepticus [3,4]. Furthermore, cEEG may be able to clarify the type of activity if the patient has stereotyped symptoms [4], such as paroxysmal movements, nystagmus, twitching, jerking, papillary hippus, or autonomic variability.

Additionally, in cases of induced coma due to high intracranial pressure or refractory status epilepticus, cEEG can be used for treatment monitoring. EEG monitoring is also suggested in conjunction with the clinical examination to assess the level of consciousness in patients requiring intravenous sedation or pharmacologically induced coma [3].

EEG monitoring could aid in grading the severity of encephalopathy associated with sepsis or other complications; a linear correlation between worsening EEG patterns and increasing mortality has been reported in the study of Young and Mantia [5]. The authors concluded that cEEG can help assess the course of the disease and the effectiveness of therapy [5]. Another clinical application of cEEG in the ICU is the recognition of delayed cerebral ischemia (DCI) due to vasospasm after subarachnoid hemorrhage [6].

Patients with acute ischemic stroke can deteriorate after the initial event due to extension of the original infarct, new infarct, or re-occlusion of a recanalized vessel. These patients may also benefit from cEEG for the detection of a new ischemic event. Furthermore, patients undergoing surgical interventions with a high risk for cerebral ischemia, such as carotid endarterectomy, could be assisted by cEEG in controlling their status during these procedures and in preventing permanent brain injury [4].

The study's objective was to assess the clinical status of comatose ICU patients utilizing cEEG and its correlation to disease prognosis. We intend to assess the impact it has on enhancing the monitoring of brain activity, finding alterations in cEEG patterns that facilitate the timely initiation of therapy or the appropriate modification of current medication. This approach could yield improved outcomes for ICU patients. In comatose patients, EEG represents a valuable neuro-prognostication tool in patients with hypoxic-ischemic brain injury (HIBI) after cardiac arrest (CA) and in traumatic brain injury (TBI) patients [6]. The presence of burst suppression or status epilepticus and the absence of EEG reactivity to external stimuli at three days after CA significantly predict poor outcome [7].

Finally, cEEG is commonly used to aid in the diagnosis of brain death, yet the recommendations for its use vary worldwide [6]. It is reported that in order to determine brain death, electrocerebral inactivity (ECI) should be demonstrated on EEG at a sensitivity of 2 μV/mm using double-distance electrodes spaced 10 cm or more apart from each other for at least 30 minutes, with intense somatosensory or audiovisual stimuli [8].

The application of cEEG in ICUs is becoming increasingly popular as a noninvasive tool for brain monitoring in critical care patients. The indications [9] for its use include the detection of nonconvulsive seizures/NCSE as well as monitoring the effect of antiepileptic therapy, the characterization of paroxysmal events that raise the suspicion of seizures, the monitoring of sedation depth, the classification of the severity of encephalopathy, the detection of cerebral ischemia, the prognostication of outcome in comatose patients, and even the detection of brain death in several countries worldwide [9].

Among critical care patients, pathological or worsening EEG patterns appear to be associated with increased morbidity and mortality. A large study of hospital records [10] found that using cEEG is linked to lower death rates in the hospital, especially for patients with subarachnoid or intracerebral hemorrhage and those with altered consciousness. In another large retrospective study conducted by Ney et al. [11], cEEG was favorably associated with inpatient survival in mechanically ventilated patients, without adding significant charges to the hospital stay when compared to routine EEG (rEEG) alone.

## Materials and methods

The protocol of this study was approved by the Scientific Committee of G. Papanikolaou Hospital (approval number 309/28.3.2024). We verbally informed the patients' relatives about the application of cEEG monitoring and obtained their verbal consent.

This study included a total of 55 patients over the age of 18 between June 2021 and December 2023. Patients had various neurological disorders, including HIBI after CA, TBI, ischemic stroke, septic shock, status epilepticus, subarachnoid or intracerebral hemorrhage, and malignant brain tumors. Subjects were screened through a cEEG study ordered during the ICU stay. The duration of monitoring was decided according to clinical indication and was scheduled for 24 hours.

The following data were collected: age, gender, medical history, sequential organ failure assessment (SOFA) score at admission, acute physiology and chronic health evaluation (APACHE) II score at admission, admission diagnosis, and comorbidities. Furthermore, the reason for undertaking EEG, the pattern of EEG, and the therapeutic strategies used in these patients were also recorded. As for the treatment modalities, we recorded the anticonvulsant drugs already prescribed to patients prior to EEG and the modifications that were made after the EEG, both in the dosage and the type of drug. We also documented the duration of mechanical ventilation and ICU length of stay (LOS). Lastly, we documented the patients' results based on the EEG diagnosis and classified them using the Glasgow Outcome Scale (GOS) for functional outcome.

cEEG recordings and data classification

**Figure 1:**
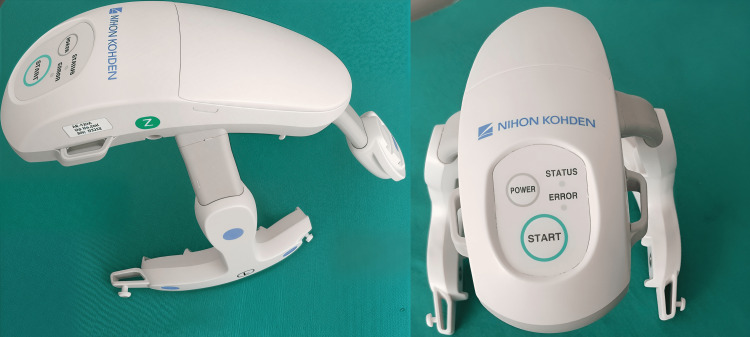
Wireless EEG headset This cEEG headset was used in our ICU, and the photo was taken by one of the authors, Dr. Georgia Vasileiadou. cEEG: continuous electroencephalogram

We used a wireless headset (CerebAir® Nihon-Kohden, Rosbach, Germany) with seven disposable electrodes attached, which connect to the headset at certain spots using a push button (Figure 1). The EEG headset [12] was placed in the intended position on the patient's head and fixed with soft bands (derivation points according to the 10/20 scheme: Cz, F3, F4, T3, T4, C3, and C4). A "Z" (zero) refers to an electrode placed on the midline sagittal plane of the skull and is present mostly for reference/measurement points. Lines [13] follow the contours of the patient's skull, and the headset's small dimensions are designed to avoid interference with other medical equipment attached to the patient's head. The flexible design of the headset [13] enables accurate electrode positioning by simply placing the pad on the patient's forehead.

After skin preparation, we affixed disposable electrodes [12] in positions P3 and P4 with a specific electrode paste to reduce electrical resistance. Data were transmitted [12] via Bluetooth® to the bedside EEG computer, displayed on the monitor, and stored with Polaris.one Software (Polaris.one, Nihon-Kohden Europe).

EEG recordings were reviewed by an expert neurology consultant. The EEG was analyzed for signs of brain problems, focal seizures, generalized seizures, status epilepticus, normal brain activity, and patterns that indicate brain death.

Definitions of EEG patterns used in the study

Lateralized periodic discharges (LPDs) [14] are stereotyped, repetitive EEG discharges that recur periodically at regular intervals of 0.5-3 Hz; they are broadly lateralized over one hemisphere, particularly over the parasagittal and temporal areas. LPDs are usually epileptiform in appearance; they appear like sharp waves/sharp waves [14] complexes ranging from 50 to 300 uV in amplitude or as blunt delta waves that recur in a stereotyped periodic fashion. They are maximal in any focal brain lesion and can sometimes be asymmetrical. They are also associated with additional EEG evidence of ipsilateral cerebral dysfunction, such as focal slowing and loss of posterior dominant rhythm [14].

Generalized periodic discharges (GPDs) are repeated generalized waveforms with relatively uniform morphology and duration, with a quantifiable interdischarge interval between consecutive waveforms and recurrence of the waveform at nearly regular intervals [15]. Global encephalopathy is suggested by the occurrence of GPDs. GPDs may arise from a wide range of encephalopathies. Common etiologies include anoxia, toxic/metabolic derangements, infections, acute neurologic injury, NCSE, and hypothermia [16].

NCS and NCSE [2] are types of seizures that show brain activity on an EEG test but usually have little or no noticeable body movements, often along with changes in awareness. The clinical features of NCSE [2] are variable and include impaired consciousness, coma, subtle facial, trunk, or limb twitches, head deviation, autonomic signs, and behavioral changes. An electrographic seizure (ESz) [2] is when the EEG shows abnormal brain activity for at least 10 seconds or any change in the pattern that clearly evolves in frequency, location, or shape. Electrographic status epilepticus (ESE) [2] is an ESz lasting for >10 minutes. An EEG is the only tool for the definite diagnosis of NCSE, as the clinical symptoms of NCSE are mild and sometimes even difficult to differentiate from normal behaviors [17].

NCSE can derive from any condition that affects the brain structure, such as cerebral anoxia, infarcts, intracranial hemorrhage (intraparenchymal/subdural/subarachnoid), neoplasm, infection, TBI, sepsis, prior history of epilepsy, metabolic abnormalities, medication overdose/toxicity (e.g., cefepime), and neurodegenerative disorders [18]. NCSE symptoms present with inattention, disorientation, confusion, abulia, abnormal eye movements (e.g., gaze deviation or nystagmus), subtle repetitive facial or distal movements of extremities, and, in more severe cases, stupor and coma [18]. Directly impacted mental status, speech disturbance, myoclonus, delirium, anxiety, agitation, extrapyramidal signs, unusual behaviors, and hallucinations are among the symptoms that have been reported and can be associated with NCSE [17].

cEEG monitoring aids in determining the need for an antiepileptic drug (AED) and the effect of prescribed AED therapy in real time. The information obtained from cEEG monitoring not only increases clinician understanding of a given patient's illness presentation but also affects treatment decisions [19].

Typical EEG features for NCSE [17] are repetitive generalized or focal spikes, polyspikes, sharp waves, and spike-and-wave or sharp-and-slow wave complexes at >2.5/second in patients without a known epileptic encephalopathy.

Statistical analysis

Frequencies and percentages were used to describe all categorical outcomes, while means and standard deviations were used for continuous measurements such as duration and age. The analysis of variance was used to check for differences in continuous measurements, and the independent samples t-test was used to analyze variations based on survival. The Pearson's chi-square test, or the Fisher's exact test, was used to examine associations between categorical variables and survival. Statistical significance was set at 0.05 in all cases, and the analysis was performed using IBM SPSS Statistics for Windows, Version 26 (Released 2019; IBM Corp., Armonk, New York).

## Results

**Figure 2:**
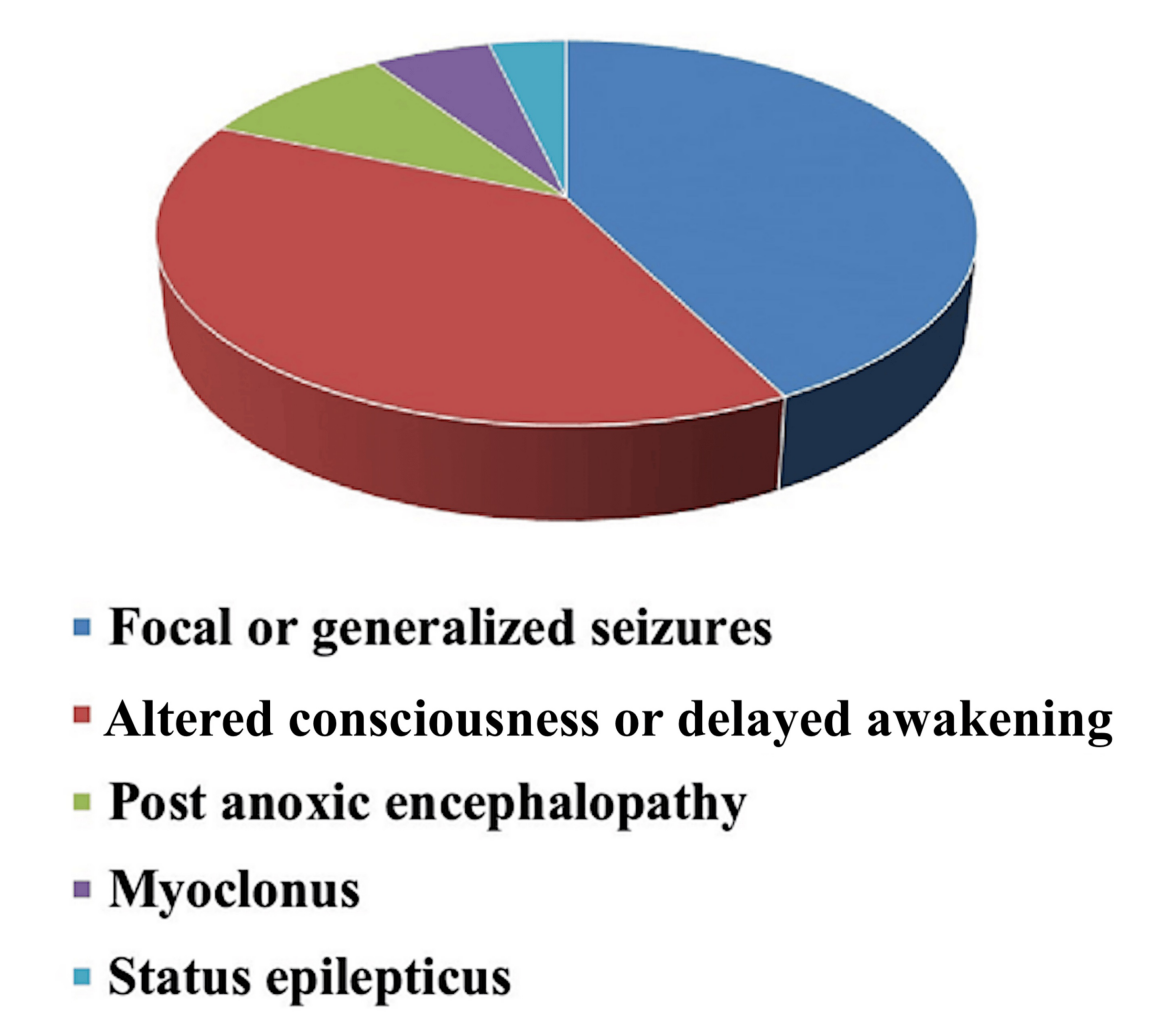
Reason for conducting cEEG in the ICU setting cEEG: continuous electroencephalogram; ICU: intensive care unit

During the study period, 55 patients underwent cEEG, with a mean age of 61 years (SD=15). A total of 34 (61.8%) patients were male. At admission, the median APACHE II score was 22 (SD=6), and the median SOFA score was 9 (SD=2.3). The median duration of mechanical ventilation was 35 days (SD=33), and the median ICU LOS was 42 days (SD=35). The ICU physicians performed cEEG recordings at a median time of 14 days (SD=18) after admission (Table 1). The majority of cEEGs (61.1%) were implemented for medical reasons, such as ischemic stroke, septic shock, or status epilepticus. Neurosurgical patients (both those with a TBI or cancer) made up 25.9% of cEEGs, while post-anoxic comatose patients made up 13%. This investigation examined cEEGs of 23 (41.8%) patients with seizures, whether focal or generalized, three (5.5%) patients who experienced myoclonus, two (3.6%) patients who presented with status epilepticus, and 22 (38.2%) patients who exhibited altered consciousness and delayed awakening (Figure 2).

**Table 1:** Patients' demographic characteristics SD: standard deviation; APACHE II: acute physiology and chronic health evaluation II; SOFA: sequential organ failure assessment; cEEG: continuous electroencephalogram; ICU: intensive care unit

Variable	Mean (SD)
Age (year), mean (SD)	61.55 (15.35)
APACHE II score, mean (SD)	22.53 (7.52)
SOFA score, mean (SD)	8.84 (2.29)
cEEG implementation after ICU admission, days, mean (SD)	13.85 (17.75)
Duration of mechanical ventilation, days, mean (SD)	34.73 (33.29)
Duration of ICU stay, days, mean (SD)	42.11 (34.82)

In 37 (67.3%) patients, cEEG was performed without sedatives. Remifentanil, an analgesic medication, was continuously infused intravenously in 18 (32.7%) of the patients. Thirty-five (63.6%) patients were already taking AEDs at the time of cEEG, whereas the most common drugs among them were levetiracetam and lacosamide.

A total of 38 (69%) patients reported cEEG diagnoses as encephalopathic, while 12 (22%) individuals had diagnoses compatible with focal seizures. Three patients presented with generalized seizures; one patient had status epilepticus, and one patient had a pattern compatible with brain death (Table 2).

**Table 2:** Diagnosis of cEEG cEEG: continuous electroencephalogram

cEEG Diagnosis	Patients, n (%)
Encephalopathic	38 (69%)
Focal seizures	12 (22%)
Generalized seizures	3 (5.4%)
Status epilepticus	1 (1.8%)
Brain death	1 (1.8%)

The diagnosis of cEEG led to several changes in the AED. In 11 (20.8%) patients, a new medication was started; in 13 (24.5%) patients, the dosage of the drug was increased; in three (5.7%) patients, the dose of the drug was decreased; and in 26 (49.1%) patients, no change in the AED was made (Table 3). Seven (12.7%) patients underwent a repeat cEEG; on this occasion, only two (3.6%) individuals received a new medication. The dosage of the medication was adjusted for the other two patients.

**Table 3:** Prescription of AEDs in correlation with the results of cEEG AEDs: antiepileptic drugs; cEEG: continuous electroencephalogram

AED Prescription	Patients, n (%)
Start AED	11 (20.8%)
Increase AED	13 (24.5%)
Decrease AED	3 (5.7%)
No change in AED	28 (49 %)

**Figure 3:**
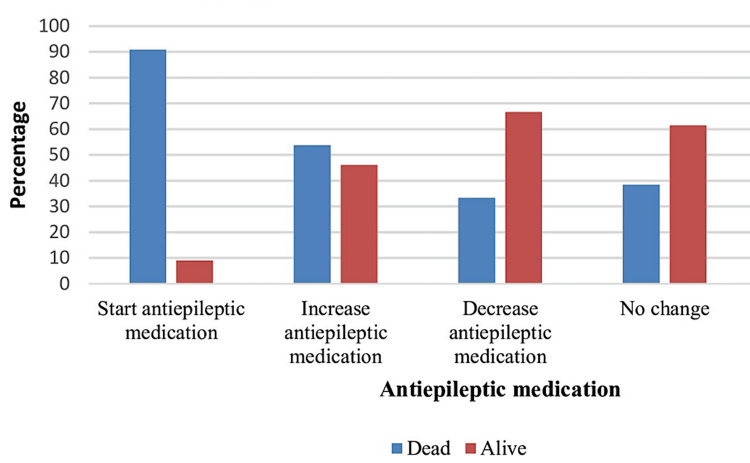
Antiepileptic medication in relation to outcome

**Figure 4:**
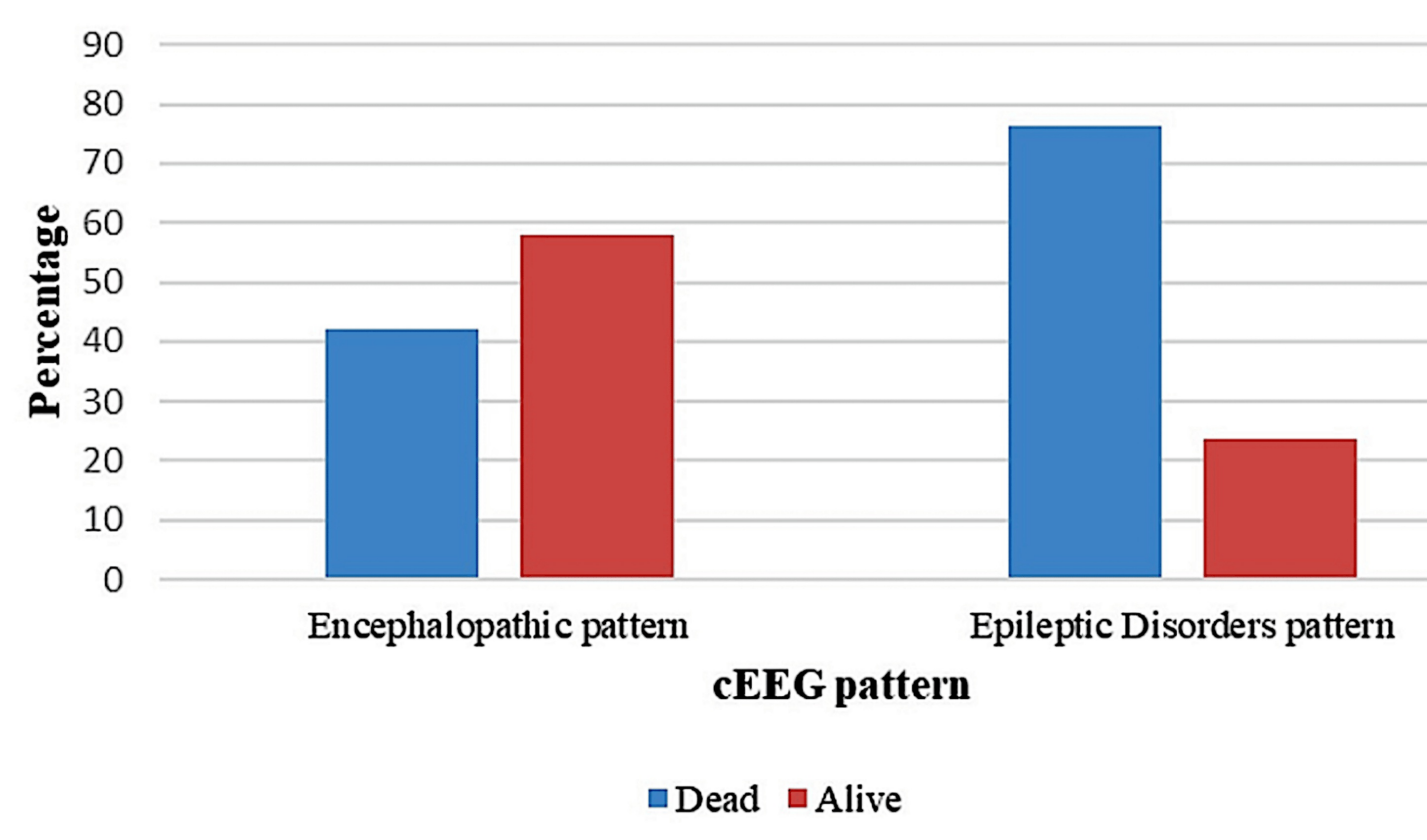
cEEG pattern in relation to outcome cEEG: continuous electroencephalogram

Patients who started AED after EEG examination had a higher mortality rate (Tables 4, 5) than patients who had already received anticonvulsant medications (Figure 3). A smaller number of patients had LPDs, but more than half of the patients had encephalopathic EEG patterns. One patient had burst suppression, and the other had ECI or ECS. Patients with epileptic disorders had a higher mortality rate (p = 0.018) in comparison with the other categories of patients (Figure 4).

**Table 4:** Prescription of AEDs in relation to outcome: calculation of marginals AEDs: antiepileptic drugs

Antiepileptic Medications	Dead	Alive	Total
Start AED	10 (90.91%)	1 (9.09%)	11 (100%)
Increase AED	7 (53.85%)	6 (46.15%)	13 (100%)
Decrease AED	1 (33.33%)	2 (66.67%)	3 (100%)
No change in AED	12 (42.86%)	16 (57.14%)	28 (100%)
Total	30 (54.55%)	25 (45.45%)	55 (100%)

**Table 5:** Prescription of AEDs in relation to outcome: cell expected values and cell chi-square values AEDs: antiepileptic drugs

Antiepileptic Medications	Dead	Alive	Chi-Square	p-value
Start AED	6 (2.67)	5 (3.20)	7.9565	.046919
Increase AED	7.09 (0.00)	5.91 (0.00)		
Decrease AED	1.64 (0.25)	1.36 (0.30)		
No change in AED	15.27 (0.70)	12.73 (0.84)		

**Figure 5:**
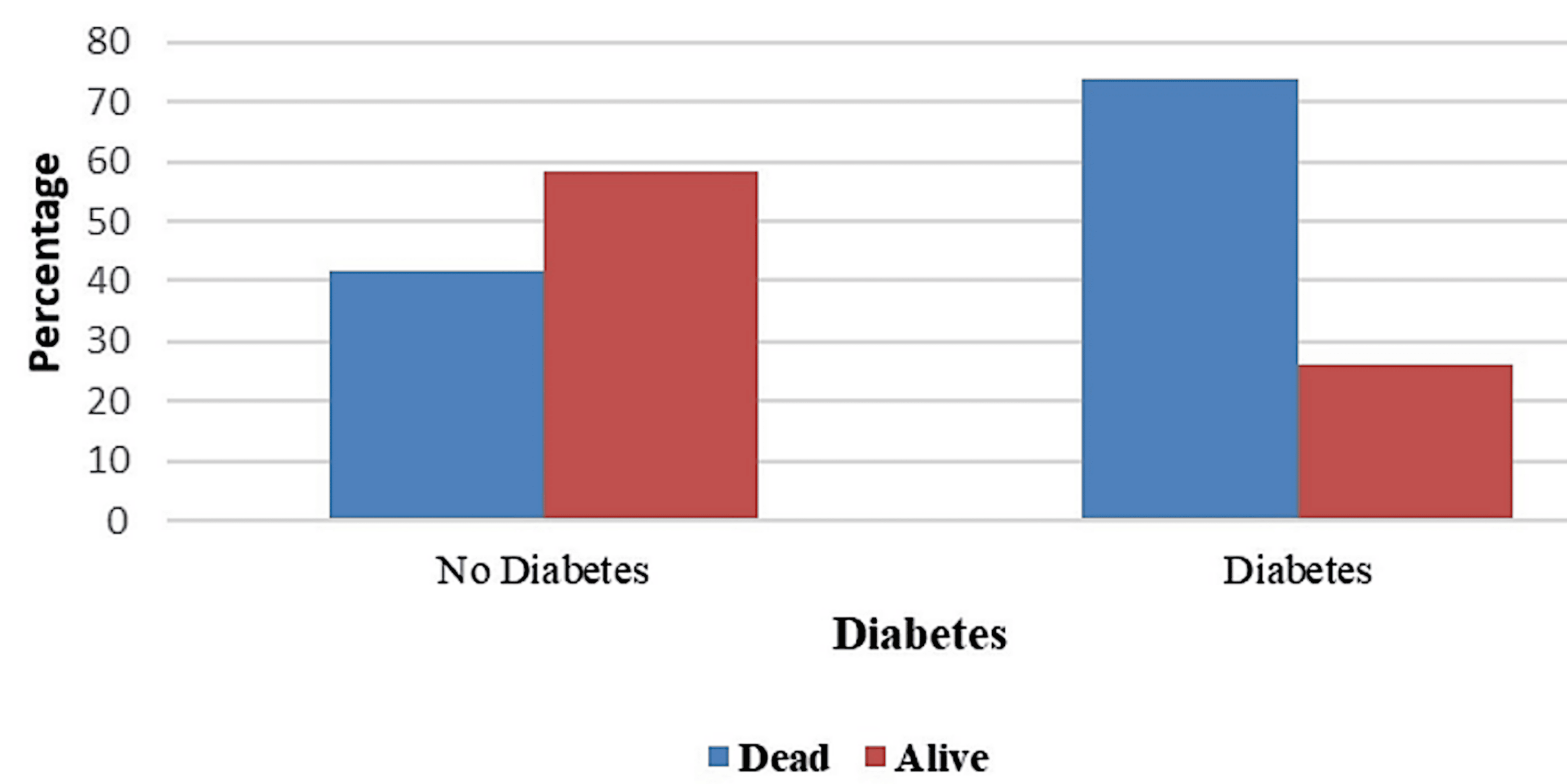
Diabetes as a comorbidity and its relation to outcome

Taking their medical comorbidities into consideration, patients diagnosed with diabetes mellitus (Tables 6, 7) were more likely to have higher mortality (Figure 5). Regarding the GOS, patients who presented with epileptic disorders of any cause had a higher mortality rate compared with those who displayed an encephalopathic pattern in the cEEG monitoring. However, this result was not significant in the statistical analysis.

**Table 6:** Diabetes mellitus in relation to outcome: calculation of marginals

Diabetes Mellitus	Dead	Alive	Total
Diabetes mellitus	14 (73.68%)	5 (26.32%)	19 (100%)
No diabetes mellitus	15 (41.67%)	21 (58.33%)	36 (100%)
Total	29 (52.73%)	26 (47.27%)	55 (100%)

**Table 7:** Diabetes mellitus in relation to outcome: cell expected values and cell chi-square values

Diabetes Mellitus	Dead	Alive	Chi-Square	p-value
Diabetes mellitus	10.02 (1.58)	8.98 (1.77)	5.1147	.024
No diabetes mellitus	18.98 (0.84)	17.02 (0.93)		

Furthermore, among survivors, regardless of statistically significant outcomes, there is a trend toward moderate and severe disability, indicating that patients with severe brain injury from any cause may continue to be functionally dependent upon ICU discharge.

## Discussion

Every critical care patient presenting with altered mental status or a variety of aberrant movements should undergo cEEG in accordance with the indications published in the Consensus Statement on Continuous EEG [3].

This observational, prospective study performed in a single general ICU setting demonstrated that cEEG did not improve the overall outcome of the patients who were monitored. However, it improved the management of patients by helping to modify their therapeutic strategies. cEEG facilitated the AED prescription when needed. Our findings approximate the findings of a study conducted by Khawaja et al. [20], who reported that the use of cEEG in the critical care setting influenced how AEDs are prescribed. However, it did not affect the outcome of discharge. The study reveals that patients initiating AEDs post-EEG examination exhibited a greater mortality rate compared to those already on anticonvulsant therapy. This finding could be attributed to a delay in conducting cEEG in a significant number of patients who were admitted with conditions unrelated to seizures or epilepsy, as indicated in the study by Khawaja et al. [20].

Although cEEG was only utilized in 0.3% of the critically ill patients' population, a study by Hill et al. [10] that included a large number of patients (more than 7000) showed that it was linked to lower in-hospital mortality. In line with our findings [21], a seizure/status epilepticus subgroup did not exhibit a lower mortality rate. This decrease is attributed to the fact that the underlying pathology and disease severity in these conditions may be the most critical determinant of outcome, often displaying poor outcomes [21]. In our study, patients with an epileptic pattern on cEEG had a higher probability of dying in the hospital or suffering severe disability.

Most patients under cEEG monitoring had impaired consciousness at admission due to various etiologies. Encephalopathic EEG patterns of different grades were recorded in a significant majority. In one-third of the patients in the study, cEEG found NCS or seizure-like activity, leading to new prescriptions or changes in their AEDs. The initiation or adjustment of treatment was determined based on the failure of improvement in their state of consciousness, necessitating a repeat evaluation for some individuals. In terms of mortality rates, functional abilities, or ICU LOS, cEEG did not produce a better clinical outcome despite its high diagnostic accuracy. While cEEG increases seizure detection and anti-seizure treatment modification in critically ill adults with impaired consciousness and no recent seizures, it is not associated with better outcomes, especially when compared to repeated rEEG [22].

The study also found that the co-occurrence of diabetes mellitus increased mortality rates, especially in patients with poor outcomes. It is known that type 2 diabetes mellitus is suggested to increase the risk of cognitive impairment and the patient's risk of developing epilepsy [23]. The hippocampus is the main source of rhythmic activity in the EEG, which reflects underlying brain network activity. A case-control study reported that the cognitive impairment in patients with diabetes mellitus was associated with changes in hippocampal volume and quantitative electroencephalograph (QEEG) changes [24]. The association between diabetes diagnosis and the outcome of patients with nephrological impairment, as demonstrated in our study, warrants additional investigation.

The use of cEEG significantly clarifies the current neurological state and prognosis of critically ill patients. The absence of normal sleep architecture and the presence of seizures are indicators of a poor outcome due to severe brain dysfunction and metabolic distress [25].

The present study was unable to analyze coma prognosis due to the relatively small sample size of patients monitored. Most studies focus on using cEEG to forecast results for patients with HIBI after CA. Even with hypothermia or sedative medication, the EEG background pattern in the first 24 hours after CA in comatose patients provides reliable information about how serious the brain damage is and helps predict outcomes in 40%-50% of patients [27].

This study has several limitations. First, data were analyzed at the time patients were discharged from the ICU, and no information about their long-term outcomes was collected. Second, the sample size was relatively small, and this could potentially influence our results. Designing a larger controlled study, incorporating additional variables, and analyzing long-term results would be beneficial.

## Conclusions

cEEG optimizes patient care and may alter treatment strategies in critically ill patients. When a seizure is being monitored, using cEEG in the ICU may help diagnose and interpret the patient's neurological condition. cEEG also influences the prescription of AEDs. The results of our investigation showed no connection between the usage of EEG and patient outcomes. More research is required to evaluate its clinical utility and enhance its diagnostic capabilities.

A cEEG in the ICU setting helps diagnose NCSE in patients with altered states of consciousness. Additionally, it can be used to prescribe AEDs and assess treatment responses for patients with NCSE. cEEG can be a helpful tool for evaluating critical care patients with TBI and HIBI following CA. However, more research is required to evaluate the outcome of patients who have received treatment and are monitored with cEEG.
